# Membrane traffic and synaptic cross-talk during host cell entry by *Trypanosoma cruzi*

**DOI:** 10.1111/j.1462-5822.2012.01818.x

**Published:** 2012-07-04

**Authors:** Claire E Butler, Kevin M Tyler

**Affiliations:** Biomedical Research Centre, Norwich School of Medicine, University of East AngliaNorwich, NR4 7TJ, UK

## Abstract

It is widely accepted that *Trypanosoma cruzi* can exploit the natural exocytic response of the host to cell damage, utilizing host cell lysosomes as important effectors. It is, though, increasingly clear that the parasite also exploits endocytic mechanisms which allow for incorporation of plasma membrane into the parasitophorous vacuole. Further, that these endocytic mechanisms are involved in cross-talk with the exocytic machinery, in the recycling of vesicles and in the manipulation of the cytoskeleton. Here we review the mechanisms by which *T. cruzi* exploits features of the exocytic and endocytic pathways in epithelial and endothelial cells and the evidence for cross-talk between these pathways.

## Introduction

*Trypanosoma cruzi*, the causative agent of Chagas disease, remains the foremost infectious cause of cardiomyopathy, a disease without vaccine and with poor therapeutic options (Steverding and Tyler, 2005; Machado *et al*., 2012). Study of *T. cruzi* as a consummate cell invader has provided insight into fundamental cellular behaviour, notably concerning repair of the plasma membrane (Reddy *et al*., 2001). *T. cruzi* is genetically heterogeneous (Zingales *et al*., 2009), however; irrespective of genotype, or whether present as metacyclic (derived from its triatomine bug vector), trypomastigote (host cell derived) or immotile amastigote (Tyler and Engman, 2001); the multiple and complex responses it elicits through interaction with surfaces of host cells ensure this parasite is infective to almost all nucleated mammalian cells – from professional phagocytes, to myocardial endothelia, to mucus coated gastric epithelia. In turn enabling a broad range of transmission routes (Yoshida *et al*., 2011).

## The parasite synapse

**Fig. 1:**
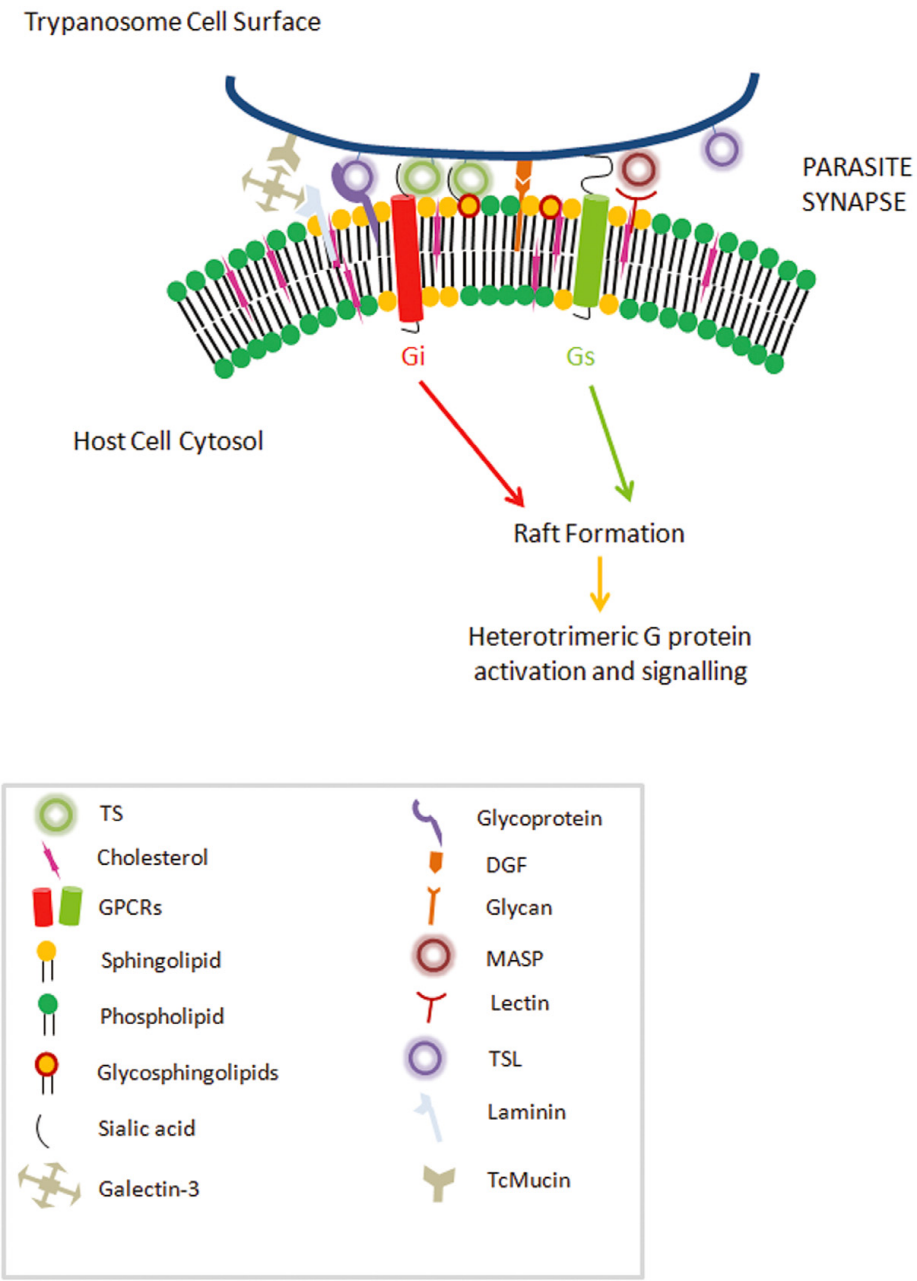
The parasite synapse initiates signalling that enables parasite entry. Cross-linking of sugars with host and parasite lectins form a lattice of glycolipid and glycoproteins on the outer leaflet where the parasite is bound. This microenvironment is rich in sterols, sphingolipids and signalling molecules such as GPCRs and inositol lipid metabolites and favours concerted signalling which leads to parasite entry.

Interaction of trypanosome and host target cell may begin even prior to attachment, with secreted parasite products of the oligopeptidase B (Caler *et al*., 1998) and parasite derived eicosanoid thromboxane A2 (Ashton *et al*., 2007) able to engender calcium mobilization. In addition, host modified substrates of the cysteine protease cruzipain, such as bradykinin, are able to initiate responses from its heterotrimeric G protein coupled receptor (GPCR) (Scharfstein *et al*., 2000). The trypanosome surface is an adherent glycocalyx, rich in glycophosphatidyl inositol (GPI)-linked glycoproteins and presumably binding firmly to host cell plasma membranes by cross-linking of glycans and lectins forming a tight synaptic junction – the parasite synapse (Fig. 1).

At the parasite synapse, trypansome mucins, GIPLs (glycosinositol phospholipids), MASPs (mucin-associated surface proteins), DGFs (dispersed gene family), TS (trans-sialidase) and TSL (trans-sialidase-like) proteins display glycans which bind host lectins and oligomerize with host integrins and other GPI-linked glycoproteins of the plasma membrane (Kleshchenko *et al*., 2004; Atwood *et al*., 2006; de Lederkremer and Agusti, 2009). Sialic acid present on host glycans (but not synthesized by trypanosomes) can also be bound and transferred within the parasite synapse by the action of the TS, while TS, TSL and DGF proteins in particular appear to be lectins in their own right and are able to bind and further cross-link host glycans on GPI-linked glycoproteins and glycosphingolipids. *T. cruzi* mucins may also bind laminin via polyvalent galectin-3 oligomers present on the host cell surface or free in the extracellular milieu thus promoting parasite-cell adhesion (Moody *et al*., 2000; Kleshchenko *et al*., 2004). A resulting lattice of this type, formed on the outer leaflet of the plasma membrane, may consequently be regarded as a type of lipid raft (Lajoie *et al*., 2009).

Lipid rafts are regions of discrete membrane heterogeneity showing increased relative order due to the alignment of the straight chain fatty acids concentrated therein. In some instances cell surface receptors are permanently localized into lipid rafts; however, other receptors may reorganize into lipid rafts after ligand binding (Zhang *et al*., 2009). Lipid rafts are sometimes regarded as signalling platforms since this environment facilitates clustering of certain receptors, transporters and signalling molecules favouring concerted signalling functions. An analogous role for lipid rafts in calcium mobilization is well established in the context of immunological synapses (Moody *et al*., 2000; Delgado *et al*., 2008).

Lipid rafts are normally enriched for sterols and sphingolipids, gangliosides such as GM1, inositol lipids and their metabolites, calcium channels, GPCRs, acylated signalling molecules such as small G proteins and tyrosine kinases, endocytosis-associated proteins such as caveolin-1 (cav1) and flotillin (flot1). Most of which have now been demonstrated to be enriched at the *T. cruzi* parasite synapse (Woolsey *et al*., 2003; Croxford *et al*., 2005; Barrias *et al*., 2007). Cytoskeletal proteins too are frequently associated with lipid rafts. In particular, WASP (Wiscott-Aldrich Syndrome Protein) is believed to regulate dynamic stability of lipid rafts by associating with cortical actin concentrating at the immunological synapse (Dupre *et al*., 2002). Also associated with actin at the plasma membrane is the protein IQGAP1 which is able to bind to microtubule tips (Fukata *et al*., 2002). Heterotrimeric G protein signalling (during *T. cruzi attachment*) may also initially depolymerize dynamic microtubules and microfilaments (Roychowdhury *et al*., 1999) which lie in immediate proximity to the inner leaflet of the parasite synapse and would initially leave a fluid pocket immediately under the rigidified synaptic membrane. Under such conditions, unpolymerized cytoskeletal elements including gamma and tyrosinated tubulin adhere to the plasma membrane, creating conditions for *de novo* polymerization of cytoskeleton and microtubule capture (Tyler *et al*., 2005).

## Plasma membrane repair and exocytosis

Parasite adhesion generates a facsimile of mechanical damage to the plasma membrane triggering plasma membrane repair via Ca^2+^-dependent exocytosis (Rodriguez *et al*., 1999; Caler *et al*., 2001). When cells undergo mechanical damage to their plasma membrane, extracellular calcium is able to enter the cytosol creating a transient and localized increase to which the cell reacts by directing lysosomes to patch and cauterize the damaged region (Reddy *et al*., 2001). Inhibition of calcium or the blocking of calcium ion channels or treatment with pertussis toxin (preventing G protein activation) results in the inhibition of this response and inhibition of trypanosome invasion (Tardieux *et al*., 1994).

An increase in intracellular calcium ions can though be achieved both by an influx of extracellular calcium after damage to the cell membrane or by mobilizing intracellular calcium stores. It is thought that *T. cruzi* uses both extracellular and intracellular calcium, and when parasite receptors cross-link appropriate host cell ligands a fast and effective invasion takes place which utilizes intracellular calcium. However, parasite mutants lacking oligopeptidase B which are deficient in the ability to mobilize calcium from intracellular stores can still invade by a slower extracellular calcium-dependent mechanism (Caler *et al*., 2000).

For exocytosis to occur, calcium must be detected inside the cell and a candidate for this function is the protein synaptotagmin VII (syt VII), a member of a family of Ca^2+^ sensors involved in vesicle exocytosis, nerve transmission and calcium-dependent membrane repair which are highly conserved in eukaryotes (Craxton, 2004). The subcellular localization of syt VII supports a role in exocytosis as separate studies have shown it to localize in the perinuclear region, the cell surface membrane and the Golgi network (Sugita *et al*., 2001; Fukuda *et al*., 2004) and have shown lysosomal targeting by colocalization with Lamp1 and after transfection (Martinez *et al*., 2000; Caler *et al*., 2001). Syt VII is essential for lysosome fusion with the cell surface membrane and is associated with calcium-dependent exocytosis, it may also be important in restricting the size of the fusion pore (Jaiswal *et al*., 2004) and in allowing lysosomes to dock with the plasma membrane (Caler *et al*., 2001). Inhibition of sytVII does reduce invasion by *T. cruzi* trypomastigotes; however, work has also shown that fibroblasts deficient in syt VII are still available for invasion (Caler *et al*., 2001; Chakrabarti *et al*., 2005).

Syt VII binds directly with Soluble NSF (*N*-ethylmaleimide-sensitive fusion protein) attachment receptors (SNAREs) regulating the docking of lysosomes to the cell surface membrane. SNAREs are made up of two groups; v-SNARES and t-SNARES. All v-SNARES are localized on vesicle membranes; vesicle associated membrane protein (VAMP) 1 and 2 (also known as synaptobrevins 1 and 2) are involved with exocytosis (Peters *et al*., 2006) while cellubrevin/VAMP3 is involved in vesicle recycling. Ti-VAMP/VAMP7 is involved in apical transport and endosome to lysosome transport (Advani *et al*., 1999). TI-VAMP/VAMP7 and the t-SNARES's SNAP-23 and syntaxin 4 have been associated with formation of the parasite vacuole and with Ca^2+^-regulated lysosomal membrane (Rao *et al*., 2004).

Construction of the parasite vacuole and retention of the parasite depends on lysosome fusion (Kleshchenko *et al*., 2004) and this relies on the movement of vesicles such as lysosomes along a microtubule network (Tyler *et al*., 2005). The requirement for a dynamic network of microtubules during *T. cruzi* invasion is reinforced by the inhibition of cell entry when microtubules are depolymerized by nocodazole, colchicine, vinblastine and taxol (Rodriguez *et al*., 1996). During invasion *T. cruzi* also appears to require kinesin as its inhibition results in the reduction of *T. cruzi* invasion events, but dynein too has been implicated in the complex lysosome motility patterns observed (Rodriguez *et al*., 1996; Tyler *et al*., 2005).

In response to parasite attachment the host cell centriole travels towards the parasite synapse (Tyler *et al*., 2005), this phenomenon of centriole docking is well characterized for the immunological synapse which is also accompanied by calcium-dependent exocytosis (Moody *et al*., 2000). Simultaneously, microtubule polymerization is observed arising from the membrane of the parasite synapse as it begins to form the nascent parasitophorous vacuole. As new microtubules are laid down around the vacuole lysosomes in the immediate vicinity migrate and fuse with the synaptic membrane facilitating formation of the vacuole. This is not a process unique to *T. cruzi*, *Toxoplasma gondii*-infected cells also exhibit translocation of the centrosome to the parasitophorous vacuole and microtubules appear to shorten around the parasitophorous vacuole (Coppens *et al*., 2006). In the absence of the host centrosome, *T. gondii* can also act as an MTOC, enabling the formation of microtubules around the parasitophorous vacuole (Romano *et al*., 2008).

In order for vesicles to reach the plasma membrane at specific focal points, the actin cortical barrier must be depolymerized. For example, at the nerve cell synapse the depolymerization of the actin-based cytoskeleton induced by the exposure to high levels of Ca^2+^ ions leads to the increase in velocity and range of movement of vesicles (Manneville *et al*., 2003). However, actin depolymerization affects the recruitment of vesicles to an injury site in an adverse manner in astrocytes due to the change in shape of the cell (Potokar *et al*., 2007). Furthermore, when cells are treated with higher concentrations of actin depolymerizing agents, this has an inhibitory effect on exocytosis (Eitzen, 2003). A similar result is also seen with secretary granule motion after cells are treated with latrunculin (Lang *et al*., 2000). This implies that the role of actin during exocytosis and *T. cruzi* cell invasion maybe more complex than just one of depolymerization to allow vesicle access.

In fact, a variety of roles for actin have been reported; depolymerization of the cortical actin barrier appears to increase invasion of *T. cruzi* (Tardieux *et al*., 1992), while the host cell membrane can ruffle around the parasite which requires the rapid localized polymerization of actin and the accumulation of actin at the parasite-cell synapse (Procopio *et al*., 1999; Mortara *et al*., 2005). Furthermore, pseudopodia-like protrusions are observed adjacent to the parasite synapse (Schenkman and Mortara, 1992). This may reflect differences in host cell type and parasite genotype, but may also be explained by the cooperation of both the exocytic and endocytic pathways.

In addition to microtubules and microfilaments, the cell cytoskeleton also comprises of intermediate filaments. The primary subunits of intermediate filaments are elongate dimers of two intertwining α-helical chains. Intermediate filaments are reversibly connected to themselves, microfilaments and microtubules by plectin which suggests that they could also be involved in exocytosis (Fuchs and Cleveland, 1998). For example, intermediate filament vimentin appears to regulate secretion in a phosphorylation-dependent manner in chromaffin cells (Quintanar, 2000). While in astrocytes intermediate filaments are crucial for efficient delivery of vesicles during exocytosis (Potokar *et al*., 2007). Keratins are intermediate filaments specific to epithelial cells. A recent study has identified a keratin-binding protein, albatross, which stabilizes the apical junction complex (AJC) and therefore aids in apical polarization and may play a role in apical sorting (Sugimoto *et al*., 2008). The silencing of cytokeratin 18 inhibits intracellular replication by *T. cruzi* which may provide a stabilizing network; however, involvement in invasion has not been demonstrated (Claser *et al*., 2008). Additionally, intermediate filaments along with microtubules stabilize the position of the *T. gondii* parasitophorous vacuole close to the nucleus (Halonen and Weidner, 1994) suggesting what may be a common strategy for intracellular parasites.

## Membrane homeostasis and endocytosis

While an explanation of microtubule dynamics and lysosomal exocytosis provides a mechanism by which materials for cell entry can be supplied, it does not explain how parasites are drawn into cells and sealed into parasitophorous vacuoles. Neither does it explain membrane ruffling which frequently accompanies parasite entry; sleeves of associated cytosol and membrane with trypomastigotes, or cups associated with amastigotes. Interestingly, in addition to the lysosome-dependent cell entry method for *T. cruzi* (Tardieux *et al*., 1992), a lysosome-independent pathway has also been proposed. This involves the formation of plasma membrane derived vesicles vacuoles which appear to account for around 50% of invasion, with another 20–30% associating with early endosomes (Woolsey *et al*., 2003). Early endosomes bud from sorting endosomes and transport internalized material to lysosomes or recycling endosomes. Early internalization vesicles were found to be covered in class I PI3 kinase products, but almost devoid of early endosome marker EEA1 indicating that parasites initially either associated with lysosomes or entered a plasma membrane-derived vacuole which then goes on to fuse with early endosomes (Woolsey and Burleigh, 2004).

Also associated with endocytosis is cav1 which acts as a scaffolding protein, coating the cytoplasmic surface of the membrane (Rothberg *et al*., 1992). Cav1 polymerizes actin inducing invagination and caveolae formation (Mundy *et al*., 2002). Caveolae have been implicated in several cases of pathogen entry, notably SV40 (Pelkmans *et al*., 2001) and intracellular protists may also use caveolae to contribute towards phagocytosis. For *T. cruzi* the recruitment of caveolin has been observed at the parasite synapse during macrophage entry (Barrias *et al*., 2007), while *Leishmania chagasi* initially resides in caveolae following phagocytosis, delaying fusion with lysosomes and promoting parasite survival (Rodriguez *et al*., 2006). Flotillins (e.g. flot1) are also raft-associated and canonically associated with another endocytic pathway which has been demonstrated to be separate to that of caveolae (Glebov *et al*., 2006) and in which formation of uncoated invaginations in the cell surface membrane depends on the co-assembly of flot1 and flot2 to promote membrane curvature (Frick *et al*., 2007). Internalization by this pathway is regulated by the src family kinase, fyn (Riento *et al*., 2009), and is associated primarily with endocytosis of GPI-linked signalling molecules (Blanchet *et al*., 2008). Very little work has been done to identify any pathogens which may exploit this pathway but flot1 recruitment has been described during entry by enteropathogenic *Escherichia coli* (Li *et al*., 2008).

An endocytic pathway not associated with the formation of lipid rafts is the clathrin-dependent pathway. Clathrin-coated pits (CCPs) do, however, require the cholesterol for stability (Rodal *et al*., 1999). Clathrin-lined invaginations are hexagonally composed appearing like honeycombs on the inside of the membrane. These structures are covered with dense clusters of receptors which when bound by the appropriate ligand induce a signalling cascade activating actin polymerization via the Arp2/3 complex to form the neck of the invagination to which the small GTPase dynamin is recruited and mediates scission of the clathrin coated vesicle (CCV) (Merrifield *et al*., 2004). Clathrin is then removed from the vesicle which then travels in an actin-dependent manner within the cytosol and can fuse with early endosomes or lysosomes depending on its cargo. CCVs can though, also bud from endomembrane compartments to traffic towards the cell plasma membrane or other endomembrane compartments.

Clathrin-dependent endocytosis is widely exploited by pathogens. *Listeria monocytogenes* for instance, utilizes surface proteins (internalins) InlA and InlB to activate two cell endocytic pathways for entry (Freitag *et al*., 2009). InlB binds the hepatocyte growth factor, Met (a tyrosine kinase), initiating signalling which results in clathrin polymerization and rearrangement of the actin cytoskeleton (Mostowy and Cossart, 2009); while, InlA binds E-cadherin in a cav1-dependent manner. In both cases the actin polymerization is Arp2/3-dependent and clathrin polymerization is vital. *T. cruzi* too, has been linked to clathrin associated cell entry via receptor mediated endocytosis of low-density lipoprotein receptors (LDLr) the depletion of which reduces parasite load. Infection with *T. cruzi* and recruitment of the LDLr to the parasite synapse appears to facilitate the interaction between lysosomes and the synaptic membrane (Nagajyothi *et al*., 2011).

Macropinocytosis occurs in response to an external stimulus resulting in the internalization of the plasma membrane into large vacuoles. The process is actin-dependent and frequently involves the ruffling of the membrane into circular protrusions and sometimes blebbing is observed (Mercer and Helenius, 2009). Macropinocytosis requires PI3 kinase for completion of the closure of macropinocytic cup (Araki *et al*., 1996; Amyere *et al*., 2000). Macropinocytosis is cholesterol-dependent (Grimmer *et al*., 2002) and PI3 kinase induces the formation of lipid rafts in macropinocytic cups and membrane ruffles which provide signalling platforms for cytoskeleton rearrangement (Mercer and Helenius, 2009). These observations are reminiscent of those observed during *T. cruzi* invasion suggesting that the possible role of macropinocytosis warrants further investigation.

## Membrane traffic and synaptic cross-talk

**Fig. 2:**
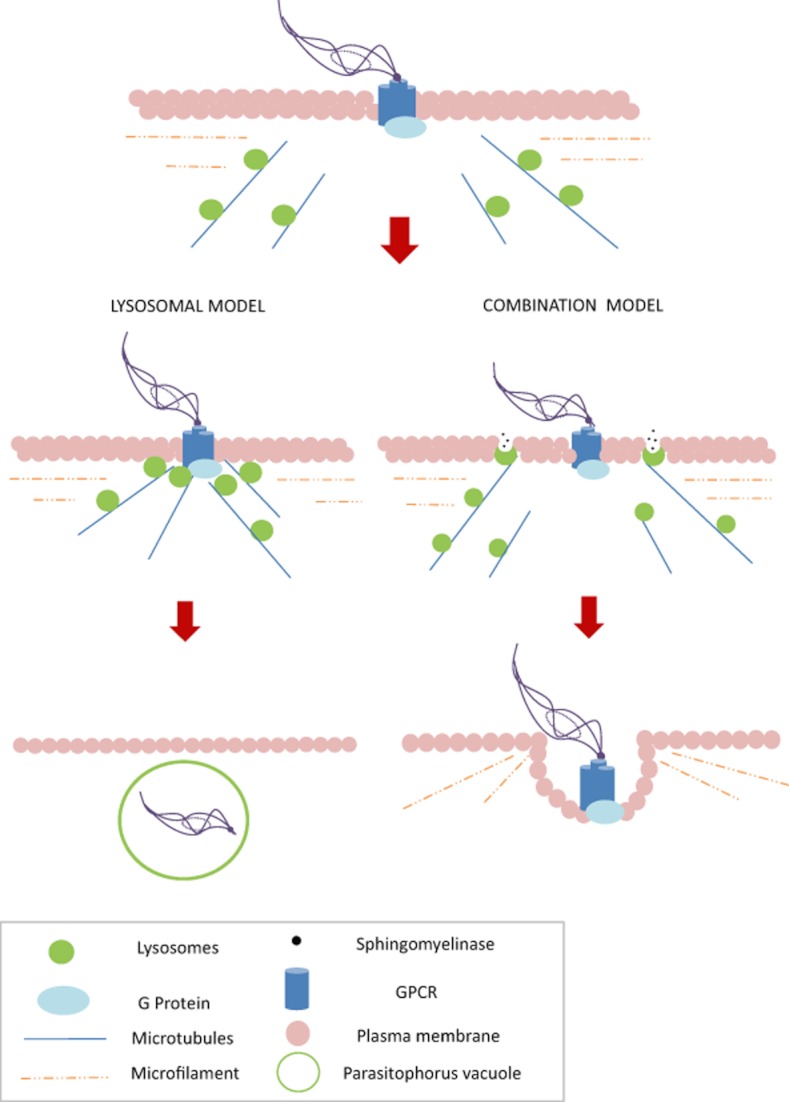
Invasion of non-phagocytic cells by *T. cruzi*. An overview of the lysosomal model for *T. cruzi* invasion where lysosomes are recruited to the plasma membrane to form the parasitophorous vacuole in a microtuble-dependent manner and a revised model where the lysosomes recruited to the plasma membrane also release acid sphingomyelinase in the vicinity of the parasite contributing to raft formation at the parasite synapse and inducing microfilament-associated endocytosis of the plasma membrane and the parasite.

Shared regulators of microtubule and microfilament dynamics accumulating at the parasite synapse suggest co-regulation of endocytic and exocytic trafficking, while cross-talk from multiple GPCRs and toll-like receptors (Aoki *et al*., 2012) may also engage with other sources of membrane such as autophagosome formation (Delgado *et al*., 2008) also implicated in cell entry (Romano *et al*., 2009). Lysosomes have previously been thought to be integral in the resealing of small pores formed by bacterial toxin (SLO); however, new evidence has emerged suggesting that endocytosis may also play a part by removing the toxins from the cell surface (Idone *et al*., 2008). Endocytosis is also more prevalent in cells which have undergone repeated mechanical or toxin-induced perforation (Reiter *et al*., 1995), and the observation that other vesicles are also seen at the site of wounding suggests that other pathways are involved (Idone *et al*., 2008). More recently, lysosomal sphingomyelinase has been implicated in the healing of plasma membrane pores caused by bacterial toxin, streptolysin O. Lysosomes secrete the enzyme acid sphingomyelinase (ASM) and inhibition of this enzyme results in the inhibition of endocytosis and membrane repair (Tam *et al*., 2010). Inhibition of ASM activity also inhibits invasion by *T. cruzi* which relies on the recruitment of lysosomes to form the parasitophorous vacuole, while induction of endocytosis can also increase the number of intracellular parasites (Fernandes *et al*., 2011). These studies provide evidence that not only are utilization of host exocytosis and endocytosis machineries during cell entry not mutually exclusive, but that they may actually be cooperative, co-dependent or even synergistic and are inherently linked (Fig. 2).
